# The p53/miR-145a Axis Promotes Cellular Senescence and Inhibits Osteogenic Differentiation by Targeting Cbfb in Mesenchymal Stem Cells

**DOI:** 10.3389/fendo.2020.609186

**Published:** 2021-01-11

**Authors:** Chao Xia, Tianyuan Jiang, Yonghui Wang, Xiaoting Chen, Yan Hu, Yanhong Gao

**Affiliations:** Department of Geriatrics, Xinhua Hospital, Shanghai Jiaotong University School of Medicine, Shanghai, China

**Keywords:** p53, microRNAs, osteogenic differentiation, mesenchymal stem cells, Cbfb, senescence

## Abstract

The osteogenic differentiation capacity of senescent bone marrow mesenchymal stem cells (MSCs) is reduced. p53 not only regulates cellular senescence but also functions as a negative regulator in bone formation. However, the role of p53 in MSCs senescence and differentiation has not been extensively explored. In the present study, we investigated the molecular mechanism of p53 in MSCs senescence and osteogenic differentiation. We found that p53 was upregulated during cellular senescence and osteogenic differentiation of MSCs respectively induced by H_2_O_2_ and BMP9. Similarly, the expression of p53-induced miR-145a was increased significantly. Furthermore, Overexpression of miR-145a in MSCs promoted cellular senescence and inhibited osteogenic differentiation. Then, we identified that p53-induced miR-145a inhibited osteogenic differentiation by targeting core binding factor beta (Cbfb), and the restoration of Cbfb expression rescued the inhibitory effects of miRNA-145a. In summary, our results indicate that p53/miR-145a axis exert its functions both in promoting senescence and inhibiting osteogenesis of MSCs, and the novel p53/miR-145a/Cbfb axis in osteogenic differentiation of MSCs may represent new targets in the treatment of osteoporosis.

## Introduction

Primary osteoporosis is a disease closely related to ageing. The population ageing trend has led to an increasing number of osteoporosis patients worldwide (1). The body is composed of numerous cells, and the ageing of the body is first manifested in the ageing of cells. In turn, cell ageing *in vitro* may in a sense reflect the process of body ageing. Although cellular senescence and ageing are not synonymous, senescence restricts the replicative life of a cell and has previously been validated as a primary mediator of aging (2). The fundamental cause of osteoporosis is the imbalance between bone formation and bone resorption, and bone formation is mainly mediated by bone marrow mesenchymal stem cells (MSCs). For senescent MSCs, the ability to undergo adipogenic differentiation was enhanced, while osteogenic differentiation was reduced, which led to a decrease in bone formation (3).

As an important tumor suppressor gene, p53 maintains the stability of the genome by causing cell cycle arrest and apoptosis under a variety of cell injury stress responses. Meanwhile, p53 has been widely implicated in cellular senescence and aging (4, 5). There are various ways to induce cellular senescence, including replicative senescence, oxidative stress, and oncogene-induced senescence, almost all of which involve changes in p53. Research shows the knock-in model of p53 showed obvious signs of ageing (6), and the deletion of an apoptosis regulator gene induced by p53 could save the loss of stem cells and decrease the signs of ageing, suggesting that the extensive apoptosis of stem cells is related to the senescence mediated by p53. In addition, p53 is involved in the regulation of bone formation (7). Osteosclerosis has been detected in p53 knockout mice, and there is also evidence that p53 regulates osteoblast differentiation through transcription factors Runx2 and Osterix (8), which are involved in osteoblast differentiation and transformation controlled by the BMP and IGF pathways. MDM2-deficient osteoblast progenitor cells have increased p53 activity and decreased level of Runx2 (9). In contrast, osteoprogenitor cells with p53 deletion showed increased proliferation and Runx2 expression, increased maturity of osteoblasts (10). These results indicate that p53 negatively regulates bone formation.

MicroRNAs (miRNAs) are a class of evolutionarily conserved, single-stranded non-coding RNA small molecules that exist in many organisms (11). They are important posttranscriptional regulators of gene expression. MiRNAs regulate gene expression by pairing with the seed complementary sequences in the 3′ untranslated region (UTR) of target mRNAs. Numerous studies have established miRNAs as powerful regulators of protein expression in physiology, including development, cell differentiation, proliferation, and apoptosis (12). Bone metabolism is also tightly regulated by the miRNAs. For example, in the initial stage of osteoblast differentiation, miRNA-29 activates the osteoblast differentiation signaling pathway by inhibiting the translation of multiple target genes, such as histone deacetylase 4 (HDAC4) (13). Another study showed that Jagged1 (JAG1), a ligand for Notch 1, as a target of miR-34a and miR-34a inhibits osteoblast differentiation by inhibiting JAG1 (14).

As a transcription factor, p53 induces the transcription expression of several miRNAs, including miR-29 and miR-34a mentioned above (15, 16). With the help of downstream miRNAs, p53 indirectly regulates miRNA target genes. Therefore, the discovery of miRNAs has added new mechanisms directing gene expression for p53. A remaining question is whether the downstream miRNAs of p53 are also involved in the regulation of cellular senescence and osteogenic differentiation of MSCs. In this study, we identified miR-145a, which was activated by p53 transcription, and studied its effects on the senescence and osteogenic differentiation of MSCs.

## Materials and Methods

### Cells

For primary mouse bone marrow MSCs (mBMSCs) culture, bone marrow cells were isolated from femurs and tibias of 6‐week‐old C57BL/6 mice and cultured in low-glucose Dulbecco’s modified Eagle’s medium (DMEM) supplemented with 10% fetal bovine serum (FBS), 1% penicillin and streptomycin. The cell suspension was plated in 10-cm dish and incubated at 37°C in a humidity incubator with 5% CO2. The medium was changed every 3 days to clear non-adherent cells. When the cells reached 80%–90% confluence, MSCs were detached with 0.25% trypsin/1 nM EDTA and passaged. After passage, MSCs received routine characterization. Except for establishing *in vitro* replicative senescent cell model, mBMSCs at three to eight passages were used in this study.

The detection of surface markers of mBMSCs was determined using flow cytometry. mBMSCs were incubated with anti-Sca-1, anti-CD44, anti-CD29, anti-CD45, or anti-CD34 for 30 min. Then the samples were examined by flow cytometry. C3H10T1/2 cells were maintained in DMEM containing 10% FBS, 1% penicillin and streptomycin.

### Osteogenic and Adipogenic Differentiation

Osteogenic differentiation of mBMSCs and C3H10T1/2 cells were induced at approximately 80% confluence in osteogenic differentiation medium (ODM) containing 10% FBS complete hight-glucose DMEM, 0.1 µM Dexamethasone, 10 mM β-Glycerophosphate and 50 µg/ml Ascorbic acid. ODM was changed every 3 days. Matrix mineralization or calcium depositions were examined with Alizarin Red staining at day 21. Cells were observed by inverted microscopy.

Adipogenic differentiation of mBMSCs was induced at approximately 80% confluence in adipogenic differentiation medium (ADM) containing 10% FBS complete hight-glucose DMEM, 0.5 mM 3-Isobutyl-1-methylxanthine (IBMX), 1 µM Dexamethasone, 10 µM Insulin, and 200 µM Indomethacin. ADM was replaced every 3 days.

### Oil Red O, Alizarin Red, Alkaline Phosphatase, and SA‐β‐Galactosidase Staining

For detection of lipid droplets, mBMSCs cultured in adipogenic medium for 2 weeks were fixed with 4% paraformaldehyde for 15 min and then stained with Oil Red O for 10 min at room temperature.

Alizarin Red and Alkaline phosphatase **(**ALP) staining were performed according to a previously described procedure (17).

SA‐β‐galactosidase staining was performed with Senescence β‐Galactosidase Staining Kit (Cell Signaling Technology, USA) following the manufacturer’s instruction. The blue‐stained cells were regarded as positive cells.

### *In Vitro* and *In Vivo* Senescent Cell Model

Oxidative stress‐induced senescence. mBMSCs and C3H10T1/2 cells were respectively incubated with 50 and 200 μM H_2_O_2_ for 2 h. Then washing with PBS, cells were maintained in fresh medium for 24 h and then harvested for mRNA and protein expression analyses.

#### Replicative Senescence

An *in vitro* replicative senescent cell model was established using long-term cultured mBMSCs.

For *in vivo* senescent cell, since we had not isolated bone marrow MSCs from ageing C57BL/6 mice successfully, *in vivo* senescent bone marrow MSCs were isolated from ageing Sprague Dawley (SD) rats (25-month-old).

### Transfection

For miRNAs transfection, the functional role of miR-145a was verified by transfecting C3H10T1/2 cells with miR-145a mimic, and its negative controls (RiboBio, China) using the Lipofectamine2000 transfection agent (Invitrogen, USA) according to the manufacturer’s instructions.

For siRNA transfection, C3H10T1/2 cells were transfected with 25nM p53 siRNA (sip53) and negative control siRNA (siNC) using Lipofectamine 2000 transfection reagent. The target sequence of siRNA was shown in **Supplemental Table S2**.

For plasmids transfection, either empty vector pcDNA3.1 (pcDNA3.1-vector) or pcDNA3.1 expressing mouse Cbfb (pcDNA3.1-Cbfb) were purchased from Sangon Biotech (Shanghai, China) and were transiently transfected in C3H10T1/2 cells using the Lipofectamine2000 transfection agent.

### Gene Expression Analysis

Total RNA was extracted with the RNAiso Plus reagent (Takara, Japan). For the mRNA analysis, total RNA was reverse-transcribed into complementary cDNA using the PrimeScript RT reagent Kit (Takara, Japan). For the miRNA analysis, the forward and reverse primers for miRNAs were designed by RiboBio Corporation (Guangzhou, China). Then, the total miRNA underwent polyA tailing and reverse transcription using the miDETECT A TrackTM miRNA qRT-PCR Starter Kit (RiboBio, China). All real-time PCR was conducted with the ABI ViiA™ 7 Real-Time PCR System (Applied Biosystems) using the SYBR Green-based real-time detection method. The following thermal settings were used: 95°C for 30 s followed by 40 cycles of 95°C for 5 s and 60°C for 30 s. The fold change was calculated with the 2^-ΔΔCT^ method and normalized to the level of the housekeeping gene GAPDH or U6, respectively. The primer sequences used in this study are listed in **Supplemental Table S1**.

### Western Blot Analysis

Whole-cell lysates were prepared on ice using cold RIPA lysis buﬀer (Beyotime, China) containing protease inhibitor (Thermo Fisher, USA). In brief, equal amounts of proteins (30 µg) were separated by sodium dodecyl sulfate-polyacrylamide gel electrophoresis (SDS–PAGE) and transferred to PVDF membranes. After incubation with the primary antibody at 4°C overnight, they were further immunoblotted with HRP-conjugated antibody at 37°C for 1 h, developed with enhanced chemiluminescence (ECL) substrate (Millipore, USA) and chemiluminescence detection by ChemiDocTM MP Imaging System (Bio-Rad, United Kingdom). Primary antibodies and dilutions used were as follows: p53 (1:1,000; Cell Signaling Technology); p21 (1:2,000; Abcam); Runx2 (1:500; Proteintech); Cbfb (1:200; Santa Cruz Biotechnology); BMP9 (1:200; Santa Cruz Biotechnology); and GAPDH (1:2000; Beyotime Biotechnology).

### Luciferase Assay

Mouse wild type or mutant Cbfb 3’-UTR regions were chemically synthesized by Sangon Biotech (Shanghai, China) and cloned into psiCHECK2 luciferase reporter plasmids. The recombinant plasmids were respectively named as, psiCHECK2‐Cbfb 3′ UTR‐WT, psiCHECK2‐Cbfb 3′ UTR‐mut. HEK293T cells were seeded in 6‐well plates. When the cells reached about 70% confluences, each recombinant plasmid (2μg) and either the miR-145a mimic or the mimic negative control (mimic NC) were co-transfected into the cells with Lipofectamine 2000. After 48 h of transfection, firefly and Renilla luciferase activities were quantified using a dual luciferase assay system (Promega, USA).

### Statistical Analysis

All data were expressed as mean ± standard deviation (SD) and analyzed using GraphPad Prism 8 (GraphPad Software Inc., USA). Differences between two groups were evaluated by unpaired two-tailed Student’s t-test. For comparison among three and more groups, one-way analysis of variance (ANOVA) was performed. All experiments were performed at least three times, and p<0.05 was considered statistically significant.

## Results

### The Characteristics of mBMSCs

**Figure 1:**
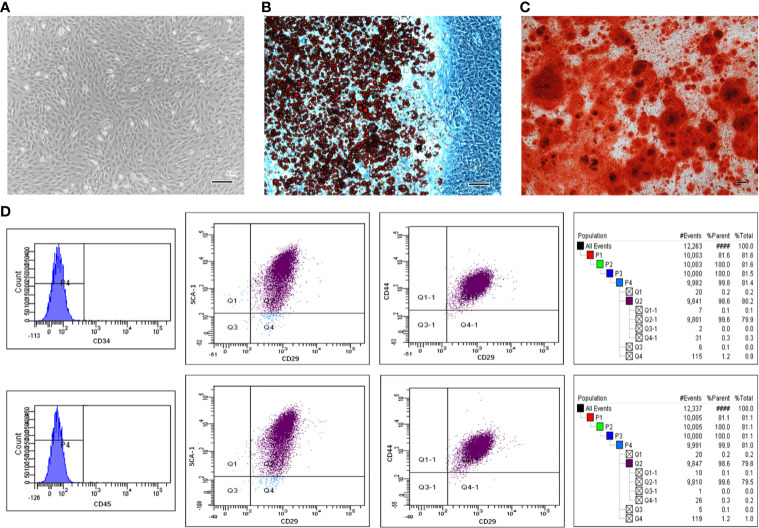
The multipotentiality and high purity of mesenchymal stem cells (MSCs) isolated from mouse bone marrow. Morphological appearance of third-passage mouse bone marrow MSCs (mBMSCs) **(A)**. Adipogenic **(B)** and osteogenic **(C)** differentiation capacity of mBMSCs. mBMSCs surface markers evaluated through flow cytometric analysis **(D)**. Bar:100μm.

By passage 3, mBMSCs were mainly observed to be bipolar spindle-like cells (**Supplemental Figure S1**). When confluence was at 90%, the cells exhibited a spiral shape (**Figure 1A**). The isolated cells showed the potential to differentiate into adipogenic and osteogenic lineages after culture in induction media. Cells contained many Oil Red-O-positive lipid globules after 2 weeks of induction with ADM (**Figure 1B**). Similarly, calcium deposits stained by Alizarin Red were detected in mBMSCs induced for 3 weeks in ODM (**Figure 1C**). mBMSCs at passage 3 were strongly positive for mBMSCs markers, including CD29, CD44, and SCA-1, and negative for CD34 and CD45 (**Figure 1D**). In summary, our results show that the mBMSCs we used in our experiments were multipotent and highly pure.

### Expression of p53 in the Process of mBMSCs Senescence and Osteogenic Differentiation

**Figure 2:**
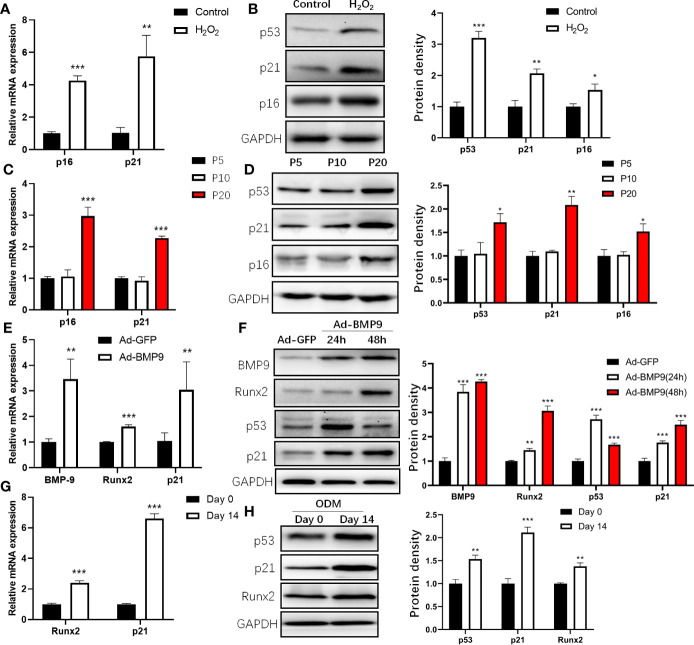
Expression of p53 during cell senescence and osteogenic differentiation of mouse bone marrow mesenchymal stem cells (mBMSCs). Expression of senescence marker, p16 and p21 determined by qRT-PCR **(A)** and Western blot **(B)** analysis during senescence of mBMSCs induced by H_2_O_2_. Expression of p16 and p21 determined by qRT-PCR **(C)** and Western blot **(D)** in long-term cultured mBMSCs. Expression of BMP9, Runx2, and p21 measured by qRT-PCR in mBMSCs after 24 h infected by Ad-BMP9 **(E)**. Western blot analysis of protein expression of p53, p21, Runx2, BMP9 in mBMSCs infected by Ad-BMP9 after 24 h and 48 h later **(F)**. Expression of p21, and Runx2 determined by qRT-PCR **(G)** and Western blot **(H)** in mBMSCs cultured in osteogenic differentiation medium (ODM) for 14 days. Results of mRNA qRT-PCR were normalized to GAPDH expression. Results are presented as the mean±SD (*p < 0.05; **p < 0.01; ***p < 0.001). Abbreviations: Runx2, Runt-related transcription factor 2; BMP9, bone morphogenetic protein 9.

Previous studies showed that H_2_O_2_ induced cell premature senescence in MSCs after stimulation (18, 19). To induce the senescence of mBMSCs, we treated mBMSCs with 50 μM H_2_O_2_ and found that the senescence markers p16 and p21 were increased (**Figure 2A**), and mBMSCs treated with H_2_O_2_ displayed significantly higher senescence-associated β-galactosidase activity than the control (**Supplemental Figure S2**). Consistent changes are also found at the protein level (**Figure 2B**). Increased levels of p21 during replicative senescence depend on p53 activation (20). Then, we examined the expression of p53 and p21 during the replicative senescence of mBMSCs by continuous long-term passage (21) and found that p21 was significantly increased after passage 20 (P20) compared with the early phase passage 5 (P5) (**Figures 2C, D**). It is possible to conclude that p53 was upregulated and activated in the process of cellular senescence induced by H_2_O_2_ or serial passage.

Then, we detected changes in p53 expression during osteogenic differentiation by inducing mBMSCs with Ad-BMP9 infection (22) and ODM. For Ad-BMP9 infection, we used a recombinant adenovirus expressing BMP9 and demonstrated that this recombinant adenovirus is capable of efficiently transducing mBMSCs (**Supplemental Figure S3**), and found p21, Runx2 mRNA increased significantly 24 h post-infection (**Figure 2E**); 48 h after infection, the change in protein levels was the same as the change in mRNA levels, but the p53 level increased 24 h post-infection and then decreased (**Figure 2F**). This may be due to the shorter half-life of wild-type p53 in normal cells (23). For mBMSCs cultured in ODM, the p21 mRNA and protein levels increased, which coincided with the change in Runx2 (**Figures 2G, H**). These results showed that the expression of p53 was upregulated both in the process of senescence and osteogenic differentiation.

### Senescent MSCs Displayed Reduced Osteogenic Differentiation Potential and p53-Knockdown Promoted Osteogenic Differentiation

**Figure 3:**
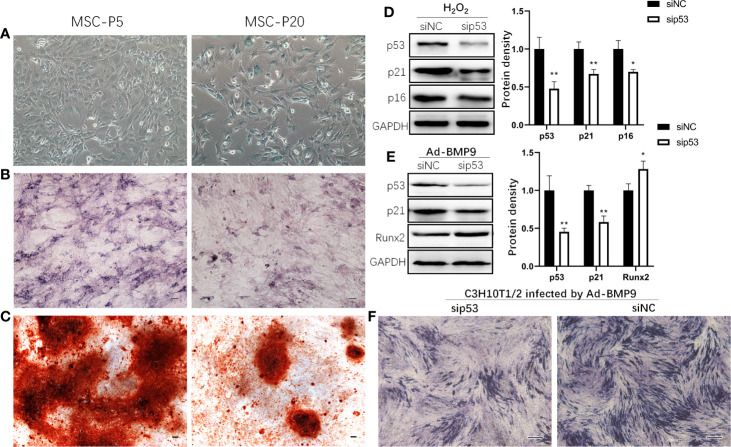
p53 knockdown inhibits senescence and promotes osteogenic differentiation of mesenchymal stem cells (MSCs). Representative microphotograph of SA-β-gal staining **(A)** performed on mouse bone marrow MSCs (mBMSCs) at passage 5 (P5) and 20 (P20). Blue staining indicates the presence of β-galactosidase activity in senescent cells. mBMSCs at P5 and P20 were cultured in osteogenic differentiation medium, and then Alkaline phosphatase (ALP) **(B)** and Alizarin Red **(C)** staining were done respectively on the 7th day and 14th day. Western blot demonstrating expression of senescence marker p16 and p21 in H_2_O_2_ induced senescent mesenchymal stem cell line C3H10T1/2 with knockdown of p53 by siRNA **(D)**. C3H10T1/2 with knockdown of p53 by siRNA were infected by Ad-BMP9. 72 h later, cells were harvested for Western blot to demonstrated expression of Runx2 **(E)** and for ALP staining **(F)**. Bar: 100μm. Results are presented as the mean±SD (*p < 0.05; **p < 0.01).

To confirm the effect of senescence on the osteogenic differentiation of MSCs, the osteogenic differentiation capabilities of MSCs were assessed by ALP and Alizarin Red staining. In comparison with mBMSCs at P5, senescent mBMSCs (P20) showed more positive cells for SA‐β‐galactosidase staining and showed significantly reduced osteogenic differentiation potential, which is indicated by the results of activated ALP evaluation and mineralization analysis (**Figures 3B, C**). Knockdown of p53 inhibited the senescence markers p16 and p21 expression in C3H10T1/2 treated with H_2_O_2_ (**Figure 3D**). And in C3H10T1/2 infected by Ad-BMP9, Runx2 expression upregulated (**Figure 3E**), and ALP activity increased (**Figure 3F**) after p53 knockdown.

### The Expression of p53-Induced miRNAs During mBMSCs Senescence and Osteogenic Differentiation

**Figure 4:**
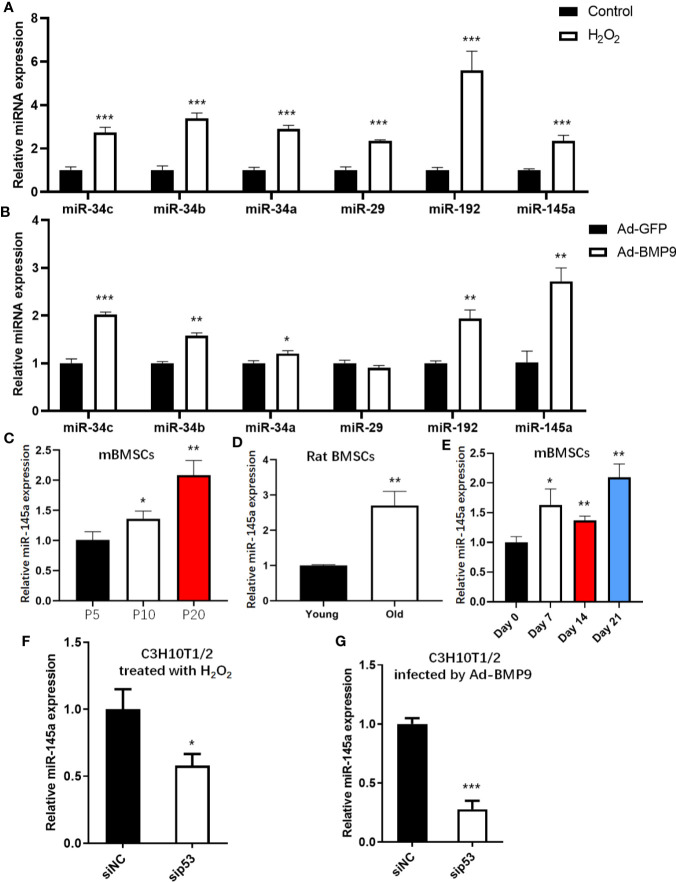
miR-145a expressed in a p53-dependent manner during senescence and osteogenic differentiation of mesenchymal stem cells (MSCs). Expression of miR-34a/b/c, miR-29, miR-192 and miR-145a were measured by qRT-PCR in MSCs treated with H_2_O_2_ **(A)** or infected by Ad-BMP9 **(B)**. Expression of miR-145a determined by qRT-PCR in long-term cultured mouse bone marrow (mBMSCs) **(C)**, in bone marrow MSCs isolated from young and ageing rats **(D)**, and in mBMSCs cultured in osteogenic differentiation medium (ODM) for 0 day, 7 days, 14 days and 21 days **(E)**. qRT-PCR demonstrating expression of miR-145a in C3H10T1/2 with knockdown of p53 by siRNA during cellular senescent induced by H_2_O_2_ **(F)** and during osteogenic differentiation induced by BMP9 **(G)**. Results of miRNA qRT-PCR were normalized to U6 snRNA expression. Results are presented as the mean±SD (*p < 0.05; **p < 0.01; ***p < 0.001). ODM, osteogenic differentiation medium.

To assess the expression levels of p53-induced miRNAs in the process of senescence and osteogenesis, we detected changes in miR-34a/b/c (24), miR-29 (16), miR-145 (25), and miR-192 (26), all of which have been validated and are well-studied p53 downstream miRNAs. These p53-induced miRNAs were upregulated in senescent mBMSCs induced by H_2_O_2_ (**Figure 4A**). Except for miR-29, other p53-induced miRNAs were upregulated after mBMSCs were infected with Ad-BMP9, and miR-145a had the largest fold-change (**Figure 4B**). To confirm the results for miR-145a shown above, we detected the expression of miR-145a in the process of replicative senescence and found that miR-145a was upregulated significantly in the late passage cells (P20) (**Figure 4C**). Additionally, since bone marrow MSCs had been successfully isolated from the ageing rats (**Supplemental Figure S4**), we measured the expression changes of miR-145a in MSCs isolated from ageing rats (25 months old) compared with those isolated from young rats (2 months old) and found miR-145a was upregulated significantly (**Figure 4D**). Moreover, we observed, on the whole, miR-145a expression was upregulated in the process of osteogenic differentiation of mBMSCs cultured in ODM (**Figure 4E**). To identify that the upregulated expression of miR-145a is p53-dependent, C3H10T1/2-sip53 were treated with H_2_O_2_ or infected by Ad-BMP9. Compared to C3H10T1/2-siNC group, miR-145a expression decreased after p53 knockdown (**Figures 4F, G**). These results showed that miR-145a expression was upregulated during mBMSCs senescence and osteogenic differentiation and the expression change was p53-dependent.

### Overexpression of miR-145a Inhibited MSCs Osteogenic Differentiation and Promoted Cellular Senescence

**Figure 5:**
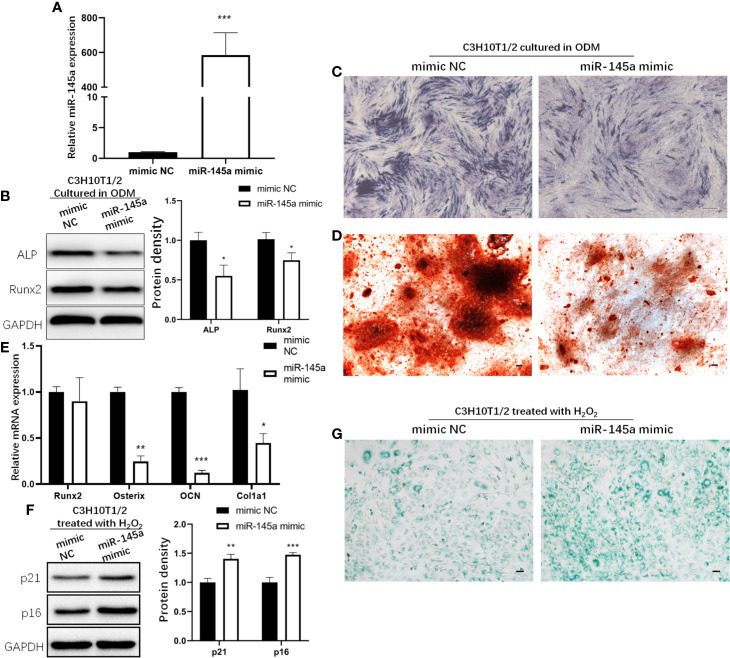
The role of miR-145a in osteogenic differentiation and cell senescence of mesenchymal stem cells (MSCs). miR-145a mimic was used to overexpress miR-145a in C3H10T1/2 by transfecting. qRT-PCR analysis of miR-145a expression on the 3rd day after transfection **(A)**. In C3H10T1/2 with miR-145a overexpression cultured in osteogenic differentiation medium (ODM): expression of Alkaline phosphatase (ALP) and Runx2 measured by Western blot on the 7th day **(B)**; ALP staining was performed on the 7th day **(C)**; Alizarin Red staining was performed on the 14th day **(D)**; the mRNA expression of osteogenic related marker Runx2, Osterix, OCN, and Col1a1 were detective by qRT-PCR **(E)** on the 14th day. C3H10T1/2 cells were transfected with miR-145a mimic. 2 days after transfection, cells were induced with 200 μM H_2_O_2_ for 2 h. After cultured in fresh medium for 24 h, cells were harvested for Western blot to detective the expression of senescence related marker p16 and p21 **(F)** and for SA-β-gal staining **(G)**. Results of miRNA and mRNA qRT-PCR were respectively normalized to U6 snRNA and GAPDH expression. Results are presented as the mean±SD (*p < 0.05; **p < 0.01; ***p < 0.001). OCN, Osteocalcin. Bar: 100μm.

miR-145a mimic was used to overexpress miR-145a in C3H10T1/2 cells, the murine mesenchymal stem cell line (27). qRT-PCR analysis of miR-145a expression confirmed a significant increase in the miR-145a overexpression group (miR-145a mimic) compared with the control group (mimic negative control, mimic NC) (**Figure 5A**). In C3H10T1/2 cells with miR-145a overexpression, the osteogenesis-associated proteins Runx2 and ALP were decreased (**Figure 5B**), and ALP and Alizarin Red staining showed the osteogenic differentiation of C3H10T1/2 cultured in ODM was inhibited (**Figures 5C, D**). Similarly, overexpression of miR-145a significantly decreased the expression of osteogenic-associated genes, including osterix, OCN, and col1a1 (**Figure 5E**). Moreover, C3H10T1/2 cells overexpressing miR-145a showed higher expression of senescence markers p21 and p16 (**Figure 5F**) and displayed significantly higher senescence-associated β-galactosidase activity than the control group (**Figure 5G**).

### miR-145a Inhibited MSCs Osteogenesis by Targeting Cbfb

**Figure 6:**
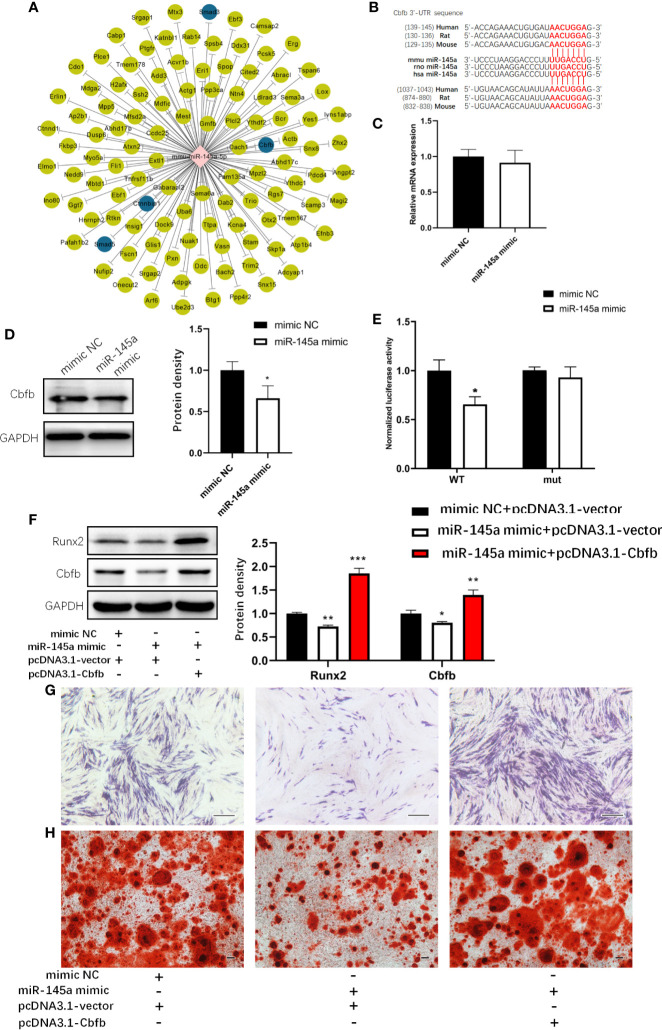
miR-145a inhibited osteogenic differentiation in C3H10T1/2 cells by targeting Cbfb. The potential miR-145a target genes predicted by TargetScan and miRDB **(A)**. Putative miR-145a binding site in Cbfb 3’-UTR **(B)**. The mRNA level **(C)** and protein level **(D)** of Cbfb measured respectively by qRT-PCR and Western blot in C3H10T1/2 overexpressed miR-145a. Luciferase reporter assay of Cbfb 3’-UTR **(E)** (Mean±SD, n=3 per group, the luciferase activity was normalized by firefly luciferase. * p < 0.05). Western blot analysis of the expression of Runx2 and Cbfb in C3H10T1/2 cells transfected with miR-145a mimic and pcDNA3.1-Cbfb and then cultured in osteogenic differentiation medium (ODM) for 3 days **(F)** and Alkaline phosphatase (ALP) staining **(G)** and Alizarin Red staining **(H)** was carried out for these cells on the 7th, 21th day, respectively. Results of mRNA qRT-PCR were normalized to GAPDH expression. Results are presented as the mean±SD (*p < 0.05; **p < 0.01; ***p < 0.001). Bar: 200μm.

Next, we examined the molecular bases behind by utilizing online databases (TargetScan and miRDB) to identify potential miR-145a target genes. The genes that overlapped in all two databases were selected to Gene Ontology (GO) analysis. Of all the predicted target genes, we found that Smad3, Cbfb, Smad5, Ctnnbip1, which was highlighted with blue colour in **Figure 6A**, are involved in osteogenic differentiation. Then we explore Smad3 and Cbfb, which had been verified as the target genes of miR-145a (28–30), given its reported role in altering bone formation and metabolism in other studies (31, 32). However, we cannot conclude that Smad3 is the target gene of miR-145a because the overexpression of miR-145a did not affect the mRNA and protein level of Smad3 in C3H10T1/2 cells (**Supplemental Figure S5A, B**). Moreover, we observed that miRNA-145a mimic did not decrease the luciferase activities of wild type Smad3-3’UTR reporter vector (**Supplemental Figure S5C, D**). Then we tested whether Cbfb is the target of miR-145a or not. An interaction between miR-145a and the complementary site within the 3′UTR of Cbfb (**Figure 6B**) has been confirmed in other studies (28, 33). **Figure 6D** shows that miR-145a targets Cbfb in C3H10T1/2 cells, given the significant reduction in Cbfb protein levels following miR-145a overexpression. No difference in Cbfb mRNA expression was found (**Figure 6C**), suggesting that miR-145a inhibits Cbfb at the level of translation in C3H10T1/2. Cbfb stabilizes Runx2 in osteoblasts by forming a complex (34). Therefore, we expected that the suppression of Cbfb by miR-145a would disturb the function of Runx2 during osteogenic differentiation. Indeed, we found a decrease in Runx2 protein levels following miR-145a overexpression in C3H10T1/2 cells (**Figure 6F**). We performed a luciferase activity assay using a reporter plasmid, psiCHECK2, in which the Cbfb 3’-UTR was cloned into the luciferase gene. Overexpression of miR-145a significantly decreased the luciferase activity, whereas a mutation in the miR-145 binding site of the Cbfb 3’-UTR abrogated the response to miR-145 (**Figure 6E**). Moreover, overexpression of Cbfb significantly reversed the inhibition of osteogenesis induced by miR-145a in C3H10T1/2 cells (**Figures 6F–H**).

## Discussion

Ageing is one of the most important risk factors for osteoporosis patients. With increasing age, the accumulation of aged MSCs in bone marrow tissue leads to the decreased ability of BMSCs to undergo osteogenic differentiation (3). The p53/p21 pathway is one of the most important signaling pathways that mediate the ageing phenomenon of most cells (35). In addition to regulating senescence, p53 negatively regulates osteogenic differentiation (36). With the discovery of miRNA, the p53/miRNAs/mRNAs axis enables the regulation scope of p53 to be more extensive and more precise (37, 38). For example, p53 can directly activate the expression of MDM2, which functions in p53 ubiquitination and degradation. However, through the p53/miR-192/MDM2 axis, p53 can negatively regulate the expression of MDM2 by activating miR-192 expression, which makes the regulation more accurate (39). In the regulation of osteogenic differentiation, p53 can directly inhibit osterix expression to negatively regulate osteogenic differentiation (8). In addition, through the transcriptional activation of miR-34a, p53 inhibits the expression of Runx2 indirectly by the p53/miR-34/Runx2 axis (10), and by transcriptional inhibition of the miR-17-92 cluster, p53 indirectly increased the expression of Smurf1 by the p53/miR-17/Smurf1 axis to ultimately inhibit osteogenic differentiation (40).

By transcriptionally regulating the expression of miRNAs, p53 participates not only in the process of cellular senescence but also in osteogenic differentiation. In this study, we found that p53 expression increased during cellular senescence and osteogenic differentiation. However, during MSCs osteogenic differentiation, p21 expression was not always consisted with p53 (**Figure 2F**). This may be partly due to the fact that p21 can be regulated independently of p53 in several situations including during cellular differentiation (41). Then, we detected the common downstream miRNAs activated by p53 transcription both in the process of senescence and osteogenic differentiation and found that miR-145a expression increased. The role of miR-145a in osteogenic differentiation has been reported previously. MiR-145a was decreased during osteogenic differentiation in human adipose-derived stem cells (ASCs), and miR-145 could suppress ASC osteogenic differentiation by suppressing FoxO1 directly (42). Consistent with this finding, another study showed that miR-145a was decreased during osteogenic differentiation in the C2C12 and MC3T3-E1 cell lines and that it suppressed osteogenic differentiation by targeting sp7 (43). However, we found that the expression level of miR-145a increased during the osteogenic differentiation of mBMSCs and C3H10T1/2. In other studies, similar results to our were reported that miR-145a was upregulated during the osteogenesis of human bone marrow-derived MSCs (44, 45). The results of miR-145a expression during osteogenic differentiation are not completely consistent, possibly due to different cell backgrounds. In addition to the target genes mentioned above (FoxO1 and SP7), Cbfb, which works as a cotranscription factor with Runx2 and thus determines the lineage of osteoblastic cells from multipotent mesenchymal cells, has also been reported as a target gene of miR-145a (28, 33). In these two studies, Cbfb was reported as a target of miR-145a in the context of osteoblast differentiation and urothelial carcinoma, respectively. Fukuda and colleagues concluded miR-145a regulates osteoblast differentiation in part *via* Cbfb. In their study, however, the mechanisms are not clarified though the authors found expression of miR-145a increased during osteoblastogenesis. In addition, the 3’-UTR sequence inserted into the luciferase reporter used for target verification was too different from that of the normal occurring 3’-UTR of Cbfb, which can be less rigorous.

Some miRNAs were downregulated in the process of osteogenic differentiation, and their function was to inhibit osteogenic differentiation. This can be explained by the fact that after the osteogenic differentiation process was initiated, the expression of miRNAs decreased under the control of certain factors so that the differentiation could be successfully completed. For example, miR-103a was downregulated during cyclic mechanical stretch (CMS)-induced osteoblast differentiation, whereas its target gene Runx2 expression was upregulated in protein level, which helps keep osteoblast differentiation running successfully (46). However, in our research, we found that miR-145a expression increased in osteogenic differentiation, though it functions as an inhibitor of osteogenic differentiation. This may be partly due to the increased p53 expression, which leads to an upregulation in miR-145a. From this point of view, p53/miR-145a axis is a mechanism for p53 to inhibit osteogenic differentiation.

Researchers have found that miR-145a overexpression promote cellular senescence in hepatic stellate cells (47). We showed that the number of senescent MSCs increased after overexpression of miR-145a. However, our study only focused on the role of the p53/miR-145a/Cbfb axis in the osteogenic differentiation, and the role of Cbfb in senescence needs further study. Interestingly, Yi-Ping Li and colleagues have reported that the deletion of Cbfb can inhibit osteogenic differentiation and enhance lipogenic differentiation (48). In accordance with this, the osteogenic differentiation ability of senescent MSCs decreased, and the lipogenic differentiation ability increased. It can be speculated that Cbfb may also play a role in the senescence of MSCs. Furthermore, it has been reported that p53 can directly bind to the Cbfb promoter region to activate its transcription in acute myeloid leukemia cells (49) and Cbfb–MYH11 fusion protein gains p53-inhibiting function *via* aberrant protein–protein interaction with HDAC8 and the p53 protein (50). Taken together, these researches suggest that p53/miR-145a/Cbfb may form a feedback loop which is similar to the p53/miR-192/MDM2 feedback loop mentioned above.

Another question needed to be addressed is the underlying molecular mechanisms of upregulation of p53 during osteogenic differentiation. Several *in vitro* studies have confirmed the role of p53 as a negative regulator in MSCs differentiation pathways (51), however, the underlying mechanisms have remained elusive. Therefore, the specific mechanism needs more in-depth study.

In conclusion, we found that p53/miR-145a axis promoted cellular senescence and inhibited the osteogenic differentiation of MSCs. These results provide a new mechanism for p53 mediated inhibition of osteogenic differentiation.

## Data Availability Statement

The original contributions presented in the study are included in the article/**Supplementary Material**. Further inquiries can be directed to the corresponding author.

## Ethics Statement

The animal study was reviewed and approved by Institutional Animal Care and Use Committee of Xinhua Hospital Affiliated to Shanghai Jiaotong University.

## Author Contributions

YG contributed to the study conception and design. CX, TJ, YW, XC, and YH performed the experiments. CX and TJ created the graphs and wrote and revised the manuscript. All authors analyzed and interpreted experimental data. All authors contributed to the article and approved the submitted version.

## Funding

This work was financially supported by the National Natural Science Foundation of China (82071575), the Science and Technology Commission of Shanghai Municipality (20ZR1435100, 18411964500, 16ZR1422000 and 18YF1415700), the Shanghai Municipal Health Commission (2020YJZX0122) and Clinical Research Plan of SHDC (SHDC2020CR3086B).

## Conflict of Interest

The authors declare that the research was conducted in the absence of any commercial or financial relationships that could be construed as a potential conflict of interest.

## Abbreviations

MSCs, Mesenchymal stem cells; miRNA, microRNA; Cbfb, Core binding factor beta; ODM, Osteogenic differentiation medium; ADM, Adipogenic differentiation medium; BMP9, Bone morphogenetic protein (BMP)-9.

## References

[1] Compston JE McClung MR Leslie WD Osteoporosis. Lancet (2019) 393(10169):364–76. 10.1016/S0140-6736(18)32112-3 30696576

[2] Gorgoulis V Adams PD Alimonti A Bennett DC Bischof O Bishop C Cellular Senescence: Defining a Path Forward. Cell (2019) 179(4):813–27. 10.1016/j.cell.2019.10.005 31675495

[3] Chen Q Shou P Zheng C Jiang M Cao G Yang Q Fate decision of mesenchymal stem cells: adipocytes or osteoblasts? Cell Death Differ (2016) 23(7):1128–39. 10.1038/cdd.2015.168 PMC4946886 26868907

[4] Hafner A Bulyk ML Jambhekar A Lahav G The multiple mechanisms that regulate p53 activity and cell fate. Nat Rev Mol Cell Biol (2019) 20(4):199–210. 10.1038/s41580-019-0110-x 30824861

[5] Ghosh S Wong SK Jiang Z Liu B Wang Y Hao Q Haploinsufficiency of Trp53 dramatically extends the lifespan of Sirt6-deficient mice. eLife (2018) 7:e32127. 10.7554/eLife.32127 29474172 PMC5825207

[6] Zhao Y Wu L Yue X Zhang C Wang J Li J A polymorphism in the tumor suppressor p53 affects aging and longevity in mouse models. eLife (2018) 7:e34701. 10.7554/eLife.34701 29557783 PMC5906094

[7] Velletri T Xie N Wang Y Huang Y Yang Q Chen X P53 functional abnormality in mesenchymal stem cells promotes osteosarcoma development. Cell Death Dis (2016) 7:e2015. 10.1038/cddis.2015.367 26775693 PMC4816167

[8] Artigas N Gamez B Cubillos-Rojas M Sanchez-de Diego C Valer JA Pons G p53 inhibits SP7/Osterix activity in the transcriptional program of osteoblast differentiation. Cell Death Differ (2017) 24(12):2022–31. 10.1038/cdd.2017.113 PMC5686339 28777372

[9] Lengner CJ Steinman HA Gagnon J Smith TW Henderson JE Kream BE Osteoblast differentiation and skeletal development are regulated by Mdm2-p53 signaling. J Cell Biol (2006) 172(6):909–21. 10.1083/jcb.200508130 PMC2063734 16533949

[10] He Y de Castro LF Shin MH Dubois W Yang HH Jiang S p53 loss increases the osteogenic differentiation of bone marrow stromal cells. Stem Cells (2015) 33(4):1304–19. 10.1002/stem.1925 PMC4376591 25524638

[11] Wang J Liu S Li J Zhao S Yi Z Roles for miRNAs in osteogenic differentiation of bone marrow mesenchymal stem cells. Stem Cell Res Ther (2019) 10(1):197. 10.1186/s13287-019-1309-7 31253175 PMC6599379

[12] Gebert LFR MacRae IJ Regulation of microRNA function in animals. Nat Rev Mol Cell Biol (2019) 20(1):21–37. 10.1038/s41580-018-0045-7 30108335 PMC6546304

[13] Tan K Peng YT Guo P MiR-29a promotes osteogenic differentiation of mesenchymal stem cells via targeting HDAC4. Eur Rev Med Pharmacol Sci (2018) 22(11):3318–26. 10.26355/eurrev_201806_15151 29917181

[14] Chen L Holmstrom K Qiu W Ditzel N Shi K Hokland L MicroRNA-34a inhibits osteoblast differentiation and in vivo bone formation of human stromal stem cells. Stem Cells (2014) 32(4):902–12. 10.1002/stem.1615 24307639

[15] Lin CP Choi YJ Hicks GG He L The emerging functions of the p53-miRNA network in stem cell biology. Cell Cycle (2012) 11(11):2063–72. 10.4161/cc.20207 PMC3368858 22580472

[16] Ugalde AP Ramsay AJ de la Rosa J Varela I Marino G Cadinanos J Aging and chronic DNA damage response activate a regulatory pathway involving miR-29 and p53. EMBO J (2011) 30(11):2219–32. 10.1038/emboj.2011.124 PMC3117645 21522133

[17] Jiang T Xia C Chen X Hu Y Wang Y Wu J Melatonin promotes the BMP9-induced osteogenic differentiation of mesenchymal stem cells by activating the AMPK/beta-catenin signalling pathway. Stem Cell Res Ther (2019) 10(1):408. 10.1186/s13287-019-1511-7 31864412 PMC6925474

[18] Choo KB Tai L Hymavathee KS Wong CY Nguyen PN Huang CJ Oxidative stress-induced premature senescence in Wharton’s jelly-derived mesenchymal stem cells. Int J Med Sci (2014) 11(11):1201–7. 10.7150/ijms.8356 PMC4166865 25249788

[19] Lin CH Li NT Cheng HS Yen ML Oxidative stress induces imbalance of adipogenic/osteoblastic lineage commitment in mesenchymal stem cells through decreasing SIRT1 functions. J Cell Mol Med (2018) 22(2):786–96. 10.1111/jcmm.13356 PMC5783884 28975701

[20] Morgner J Ghatak S Jakobi T Dieterich C Aumailley M Wickstrom SA Integrin-linked kinase regulates the niche of quiescent epidermal stem cells. Nat Commun (2015) 6:8198. 10.1038/ncomms9198 26349061 PMC4569844

[21] Matsuda S Nakagawa Y Kitagishi Y Nakanishi A Murai T Reactive Oxygen Species, Superoxide Dimutases, and PTEN-p53-AKT-MDM2 Signaling Loop Network in Mesenchymal Stem/Stromal Cells Regulation. Cells (2018) 7(5):36. 10.3390/cells7050036 PMC5981260 29723979

[22] Lamplot JD Qin J Nan G Wang J Liu X Yin L BMP9 signaling in stem cell differentiation and osteogenesis. Am J Stem Cells (2013) 2(1):1–21. https://www.ncbi.nlm.nih.gov/pmc/articles/PMC3636726/ 23671813 PMC3636726

[23] Muller PA Vousden KH p53 mutations in cancer. Nat Cell Biol (2013) 15(1):2–8. 10.1038/ncb2641 23263379

[24] Rokavec M Li H Jiang L Hermeking H The p53/miR-34 axis in development and disease. J Mol Cell Biol (2014) 6(3):214–30. 10.1093/jmcb/mju003 24815299

[25] Sachdeva M Zhu S Wu F Wu H Walia V Kumar S p53 represses c-Myc through induction of the tumor suppressor miR-145. Proc Natl Acad Sci USA (2009) 106(9):3207–12. 10.1073/pnas.0808042106 PMC2651330 19202062

[26] Georges SA Biery MC Kim SY Schelter JM Guo J Chang AN Coordinated regulation of cell cycle transcripts by p53-Inducible microRNAs, miR-192 and miR-215. Cancer Res (2008) 68(24):10105–12. 10.1158/0008-5472.CAN-08-1846 19074876

[27] Guan X Gao Y Zhou J Wang J Zheng F Guo F miR-223 Regulates Adipogenic and Osteogenic Differentiation of Mesenchymal Stem Cells Through a C/EBPs/miR-223/FGFR2 Regulatory Feedback Loop. Stem Cells (2015) 33(5):1589–600. 10.1002/stem.1947 25641499

[28] Fukuda T Ochi H Sunamura S Haiden A Bando W Inose H MicroRNA-145 regulates osteoblastic differentiation by targeting the transcription factor Cbfb. FEBS Lett (2015) 589(21):3302–8. 10.1016/j.febslet.2015.09.024 26450370

[29] Yu FY Xie CQ Sun JT Peng W Huang XW Overexpressed miR-145 inhibits osteoclastogenesis in RANKL-induced bone marrow-derived macrophages and ovariectomized mice by regulation of Smad3. Life Sci (2018) 202:11–20. 10.1016/j.lfs.2018.03.042 29577879

[30] Huang W Huang C Ding H Luo J Liu Y Fan R Involvement of miR-145 in the development of aortic dissection via inducing proliferation, migration, and apoptosis of vascular smooth muscle cells. J Clin Lab Anal (2019) 34(1):e23028. 10.1002/jcla.23028 31489719 PMC6977357

[31] Huang Y Zheng Y Jia L Li W Long Noncoding RNA H19 Promotes Osteoblast Differentiation Via TGF-beta1/Smad3/HDAC Signaling Pathway by Deriving miR-675. Stem Cells (2015) 33(12):3481–92. 10.1002/stem.2225 26417995

[32] Qin X Jiang Q Matsuo Y Kawane T Komori H Moriishi T Cbfb regulates bone development by stabilizing Runx family proteins. J Bone Mineral Res Off J Am Soc Bone Mineral Res (2015) 30(4):706–14. 10.1002/jbmr.2379 25262822

[33] Ostenfeld MS Bramsen JB Lamy P Villadsen SB Fristrup N Sorensen KD miR-145 induces caspase-dependent and -independent cell death in urothelial cancer cell lines with targeting of an expression signature present in Ta bladder tumors. Oncogene (2010) 29(7):1073–84. 10.1038/onc.2009.395 19915607

[34] Lim KE Park NR Che X Han MS Jeong JH Kim SY Core binding factor beta of osteoblasts maintains cortical bone mass via stabilization of Runx2 in mice. J Bone Mineral Res Off J Am Soc Bone Mineral Res (2015) 30(4):715–22. 10.1002/jbmr.2397 PMC7363154 25358268

[35] Rufini A Tucci P Celardo I Melino G Senescence and aging: the critical roles of p53. Oncogene (2013) 32(43):5129–43. 10.1038/onc.2012.640 23416979

[36] Wang X Kua HY Hu Y Guo K Zeng Q Wu Q p53 functions as a negative regulator of osteoblastogenesis, osteoblast-dependent osteoclastogenesis, and bone remodeling. J Cell Biol (2006) 172(1):115–25. 10.1083/jcb.200507106 PMC2063539 16380437

[37] He X He L Hannon GJ The guardian’s little helper: microRNAs in the p53 tumor suppressor network. Cancer Res (2007) 67(23):11099–101. 10.1158/0008-5472.CAN-07-2672 18056431

[38] Hermeking H MicroRNAs in the p53 network: micromanagement of tumour suppression. Nat Rev Cancer (2012) 12(9):613–26. 10.1038/nrc3318 22898542

[39] Pichiorri F Suh SS Rocci A De Luca L Taccioli C Santhanam R Downregulation of p53-inducible microRNAs 192, 194, and 215 impairs the p53/MDM2 autoregulatory loop in multiple myeloma development. Cancer Cell (2010) 18(4):367–81. 10.1016/j.ccr.2010.09.005 PMC3561766 20951946

[40] Liu W Qi M Konermann A Zhang L Jin F Jin Y The p53/miR-17/Smurf1 pathway mediates skeletal deformities in an age-related model via inhibiting the function of mesenchymal stem cells. Aging (2015) 7(3):205–18. 10.18632/aging.100728 PMC4394731 25855145

[41] Macleod KF Sherry N Hannon G Beach D Tokino T Kinzler K p53-dependent and independent expression of p21 during cell growth, differentiation, and DNA damage. Genes Dev (1995) 9(8):935–44. 10.1101/gad.9.8.935 7774811

[42] Hao W Liu H Zhou L Sun Y Su H Ni J MiR-145 regulates osteogenic differentiation of human adipose-derived mesenchymal stem cells through targeting FoxO1. Exp Biol Med (2018) 243(4):386–93. 10.1177/1535370217746611 PMC6022928 29249185

[43] Jia J Tian Q Ling S Liu Y Yang S Shao Z miR-145 suppresses osteogenic differentiation by targeting Sp7. FEBS Lett (2013) 587(18):3027–31. 10.1016/j.febslet.2013.07.030 23886710

[44] Malcolm DW Benoit DS Nanoparticle-mediated delivery of siRNAs modulates mesenchymal stem cell differentiation. Front Bioeng Biotechnol (2016) Conference Abstract 10th World Biomaterials Congress. 10.3389/conf.FBIOE.2016.01.01507

[45] Baglio SR Devescovi V Granchi D Baldini N MicroRNA expression profiling of human bone marrow mesenchymal stem cells during osteogenic differentiation reveals Osterix regulation by miR-31. Gene (2013) 527(1):321–31. 10.1016/j.gene.2013.06.021 23827457

[46] Zuo B Zhu J Li J Wang C Zhao X Cai G microRNA-103a functions as a mechanosensitive microRNA to inhibit bone formation through targeting Runx2. J Bone Mineral Res Off J Am Soc Bone Mineral Res (2015) 30(2):330–45. 10.1002/jbmr.2352 25195535

[47] Yang J Lu Y Yang P Chen Q Wang Y Ding Q MicroRNA-145 induces the senescence of activated hepatic stellate cells through the activation of p53 pathway by ZEB2. J Cell Physiol (2019) 234(5):7587–99. 10.1002/jcp.27521 30479019

[48] Wu M Wang Y Shao JZ Wang J Chen W Li YP Cbfbeta governs osteoblast-adipocyte lineage commitment through enhancing beta-catenin signaling and suppressing adipogenesis gene expression. Proc Natl Acad Sci USA (2017) 114(38):10119–24. 10.1073/pnas.1619294114 PMC5617241 28864530

[49] Morita K Noura M Tokushige C Maeda S Kiyose H Kashiwazaki G Autonomous feedback loop of RUNX1-p53-CBFB in acute myeloid leukemia cells. Sci Rep (2017) 7(1):16604. 10.1038/s41598-017-16799-z 29192243 PMC5709397

[50] Qi J Singh S Hua WK Cai Q Chao SW Li L HDAC8 Inhibition Specifically Targets Inv(16) Acute Myeloid Leukemic Stem Cells by Restoring p53 Acetylation. Cell Stem Cell (2015) 17(5):597–610. 10.1016/j.stem.2015.08.004 26387755 PMC4636961

[51] Molchadsky A Rivlin N Brosh R Rotter V Sarig R p53 is balancing development, differentiation and de-differentiation to assure cancer prevention. Carcinogenesis (2010) 31(9):1501–8. 10.1093/carcin/bgq101 20504879

[52] Xia C Jiang TY Wang YH Chen XT Hu Y Gao YH The Role of p53-induced miR-145a in Senescence and Osteogenesis of Mesenchymal Stem Cells (2020). Available at: https://www.researchsquare.com/article/rs-17497/v1 (Accessed March 17, 2020).

