# Supplementary material for: The p53/miR-145a Axis Promotes Cellular Senescence and Inhibits Osteogenic Differentiation by Targeting Cbfb in Mesenchymal Stem Cells
Source: Front Endocrinol (Lausanne). 2021 Jan 11;11:609186. doi: 10.3389/fendo.2020.609186 (PMC7829338; doi:10.3389/fendo.2020.609186)
Supplement: Supplementary file 1 [file DataSheet_1.docx]

**Supplementary Figures**


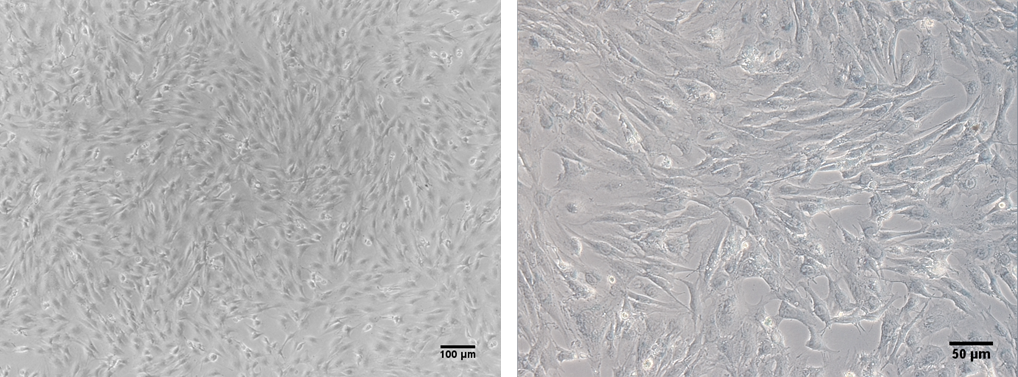


**Figure S1**. The cell morphology of MSCs isolated from mouse bone marrow under the inverted phase microscope.


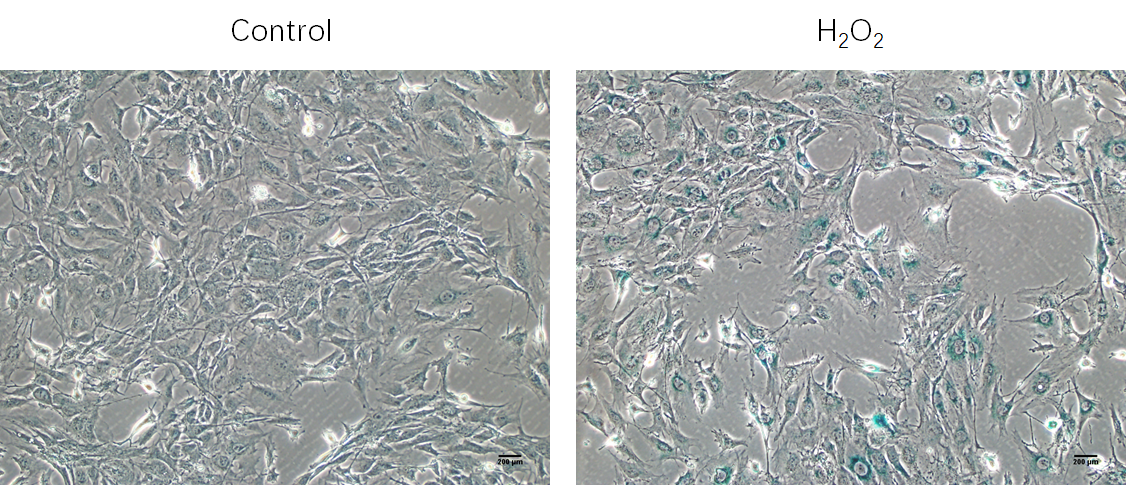


**Figure S2**. SA‐β‐galactosidase staining for ageing mBMSCs induced by H_2_O_2_


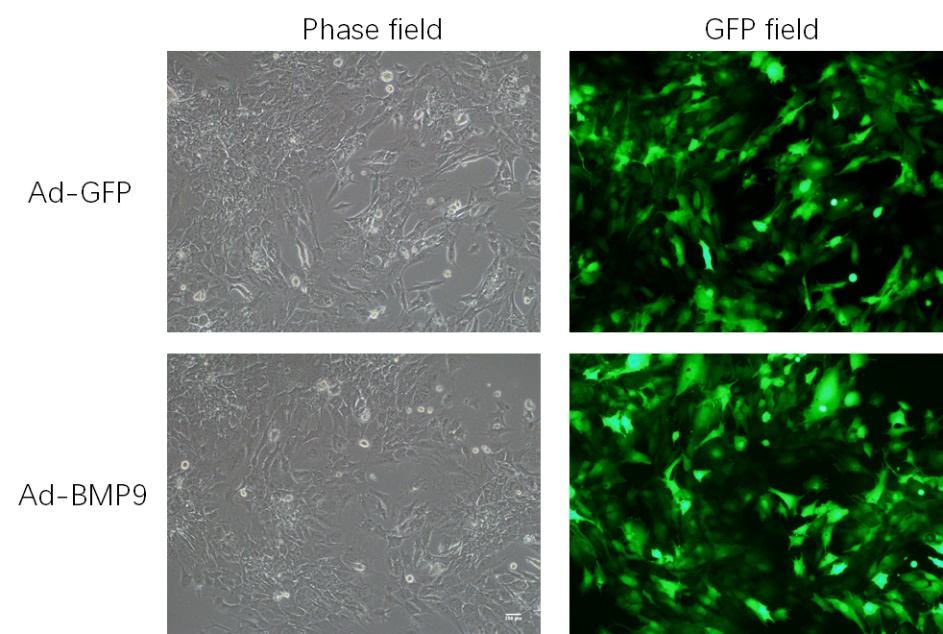


**Figure S3**. The efficiency of recombinant adenovirus infection for mBMSCs.


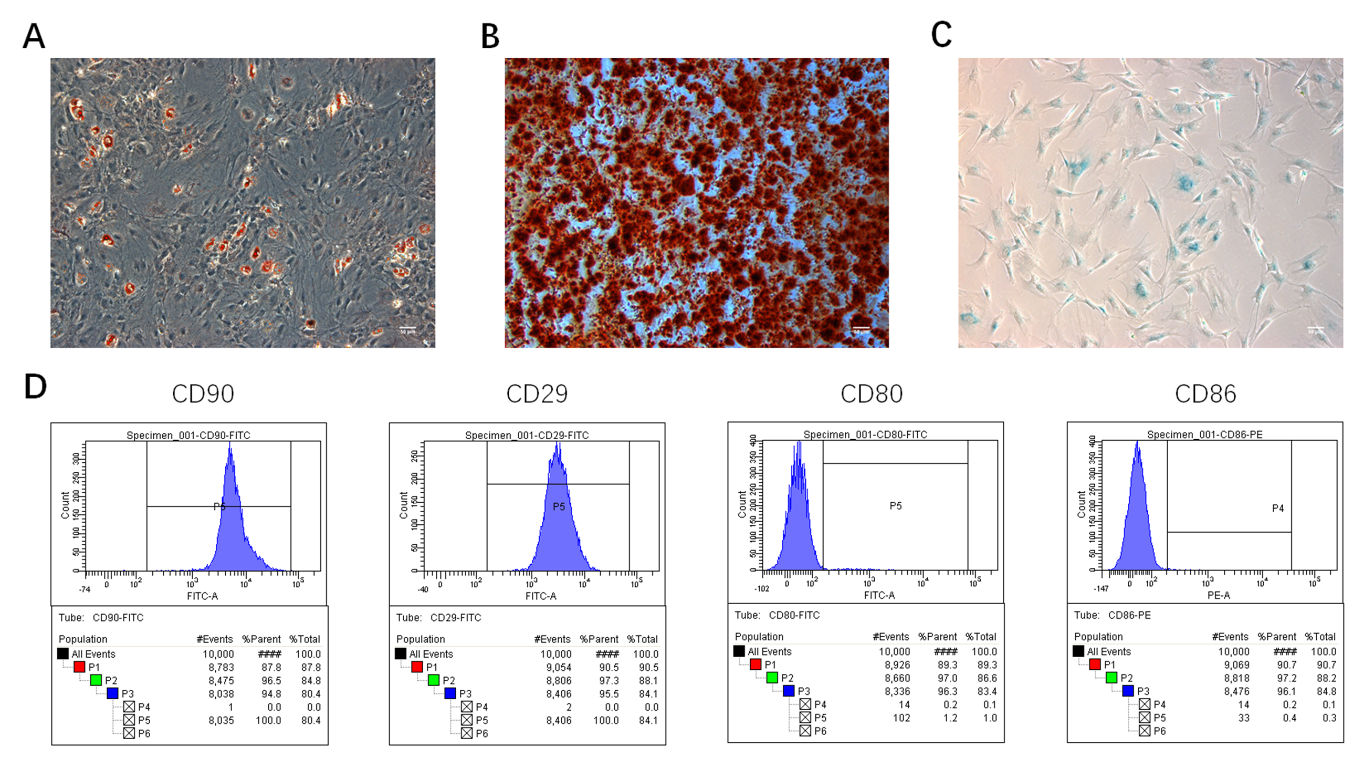


**Figure S4**. The characterization of MSCs isolated from rats bone marrow. Adipogenic (A) and osteogenic (B) differentiation capacity of rat bone marrow MSCs. MSCs surface markers evaluated through flow cytometric analysis (D). SA‐β‐galactosidase staining for MSCs isolated from bone marrow of ageing rats (C).


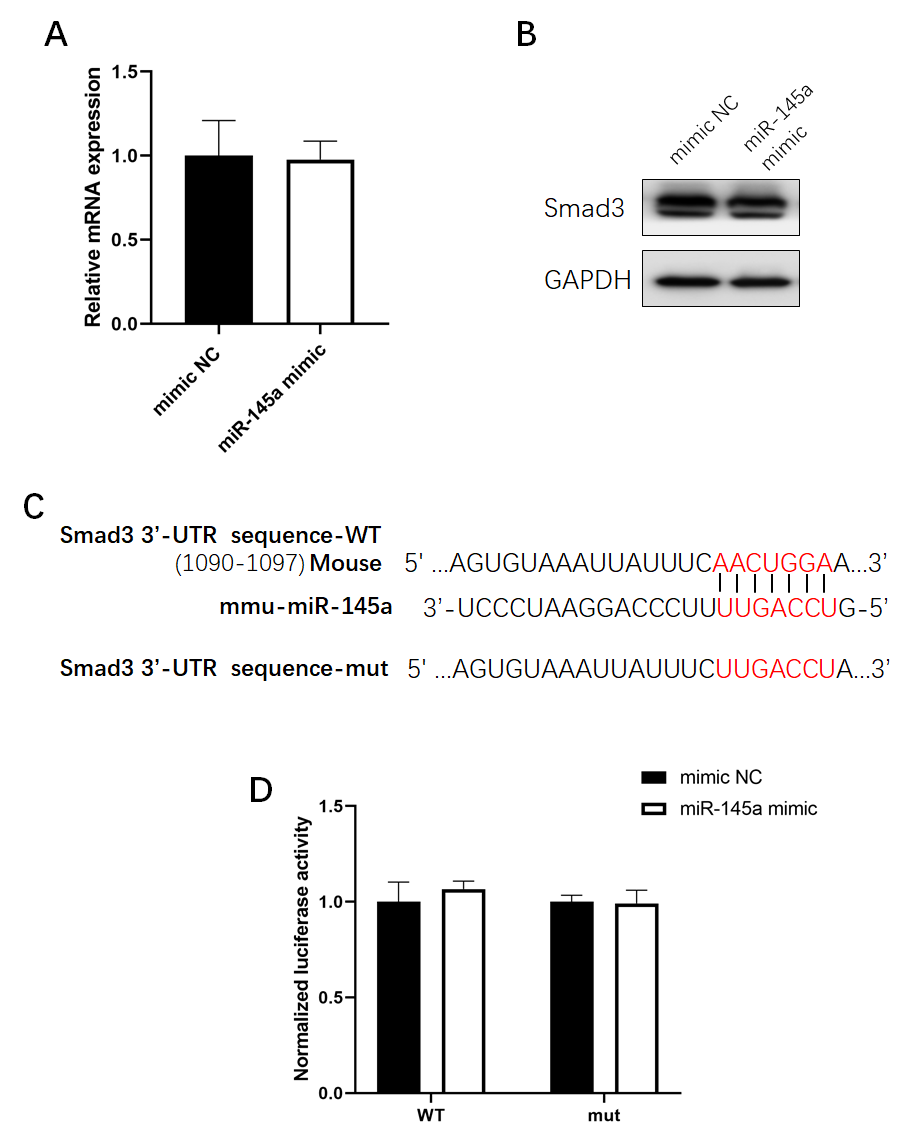


**Figure S5**. Smad3 may not be a target gene of miR-145a. The mRNA level (A) and protein level (B) of Smad3 detected respectively by qRT-PCR and western blot analysis in C3H10T1/2 cells overexpressed miR-145a. Putative miR-145a binding site in Smad3 3’-UTR (C). Luciferase reporter assay of Smad3 3’-UTR (D).

| **Gene** | **Forward sequence (5’—3’)** | **Reverse sequence (5’—3’)** |
| --- | --- | --- |
| Mouse-p53 | CCCCTGTCATCTTTTGTCCCT | AGCTGGCAGAATAGCTTATTGAG |
| Mouse-p21 | CCTGGTGATGTCCGACCTG | CCATGAGCGCATCGCAATC |
| Mouse-Runx2 | GACTGTGGTTACCGTCATGGC | ACTTGGTTTTTCATAACAGCGGA |
| Mouse-BMP9 | CCCTGGGATTGTCTGGAGC | AGGTTAAGGCTGCGTAGGAAA |
| Mouse-osterix | GGAAAGGAGGCACAAAGAAGC | CCCCTTAGGCACTAGGAGC |
| Mouse-OCN | CTGACCTCACAGATCCCAAGC | TGGTCTGATAGCTCGTCACAAG |
| Mouse-Col1a1 | GCTCCTCTTAGGGGCCACT | ATTGGGGACCCTTAGGCCAT |
| Mouse-Cbfb | ACAAACACCTAGCCGGGAATA | GCTGTGAAACTCTCACCTCCATT |
| Mouse-GAPDH | AGGTCGGTGTGAACGGATTTG | GGGGTCGTTGATGGCAACA |
| Rat-p53 | CAGCTTTGAGGTTCGTGTTTGT | ATGCTCTTCTTTTTTGCGGAAA |
| Rat-p21 | TGGACAGTGAGCAGTTGAGC | ACACGCTCCCAGACGTAGTT |
| Rat-GAPDH | TGATTCTACCCACGGCAAGTT | TGATGGGTTTCCCATTGATGA |

**Table S1.** Primers used for real-time PCR

**Table S2.** Sequences for siRNA

| **siRNA** | **target sequence (5’--3’)** |
| --- | --- |
| siNC | UGGUUUACAUGUUGUGUGA |
| sip53 | AAGTCTGTTATGTGCACGTAC |
